# Relationship between tei index and left ventricular geometric patterns in a hypertensive population: a cross-sectional study

**DOI:** 10.1186/1476-7120-9-21

**Published:** 2011-07-28

**Authors:** Kamilu M Karaye

**Affiliations:** 1 Department of Medicine, Bayero University and Aminu Kano Teaching Hospital, Kano, Nigeria

## Abstract

**Background:**

The relationship between Tei Index (TI) and left ventricular (LV) geometric patterns has not been previously well described. The present study therefore set out to describe the nature of this relationship if any, and to also assess whether a relationship exists between the geometric patterns and LV ejection fraction (LVEF) so as to establish a basis for comparison.

**Methods:**

The study was carried out in the echocardiography laboratory of Aminu Kano Teaching Hospital (AKTH) in Kano, North-Western Nigeria. The study was cross-sectional in design. Hypertensive subjects referred for echocardiography to AKTH were serially recruited from October 2008 to September 2009. TI was defined as the sum of isovolumic contraction and relaxation times divided by the ejection time, and values of LV TI < 0.40 were considered normal, while higher values were considered abnormal. Four patterns of LV geometry (normal, concentric remodelling, concentric LV hypertrophy and eccentric LV hypertrophy) were determined from the LV mass index and LV relative wall thickness as previously described. Binary logistic regression models and Pearson's Correlation (r) Coefficient were used to analyse the associations between TI or LVEF and a number of variables.

**Results:**

A total of 142 subjects were recruited into the study. The prevalence of abnormal TI (26.8%; 38 persons) in the total population was lower than that of reduced LV ejection fraction (< 50%) (38.0%; 54 persons) (p = 0.335). There was no association between any LV geometric pattern and abnormal TI. However, there was significant relationship between the geometric patterns and low LVEF (< 50%); tested in a binary logistic regression model. HR was a significant predictor of TI with regression coefficient of -0.218, 95% confidence interval (CI) of -0.005 - < -0.001 and p-value of 0.011. Similarly, HR was the only variable that significantly predicted abnormal TI in a binary logistic regression model with an odds ratio of 1.058 (95% CI = 1.002-1.118; p = 0.044), and also the only variable that correlated with TI significantly (r = -0.212; p-value = 0.014).

**Conclusion:**

This study has found that LV geometric patterns and LVEF were not associated with TI in hypertensives, but there was strong association between LV geometric patterns and LVEF. TI was found to be dependent on HR.

## Background

**Figure 1:**
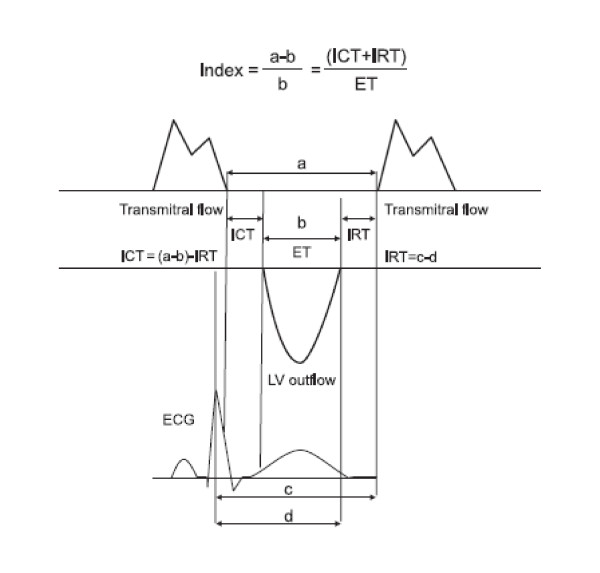
**How to calculate Tei Index**. TI is defined by the equation ('a'-'b')/'b', where 'a' represents the interval between cessation and onset of mitral inflow, and 'b' represents the ejection time (ET) of the left ventricular outflow. Isovolumic relaxation time (IRT) is measured by subtracting 'd', the interval from the peak of the R wave on the ECG to the end of ejection time, from the interval 'c' between the R wave and the onset of mitral inflow. Isovolumic contraction time (ICT) is obtained by subtracting IRT from 'a'-'b'.

Myocardial Performance Index or Tei Index (TI) was first described by Tei and Colleagues in 1995 as a "simple and reproducible Doppler index of combined systolic and diastolic myocardial performance in patients with primary myocardial systolic dysfunction" [1]. TI has since been studied in several other cardiac disorders including systemic hypertension, heart failure of various aetiologies and acute myocardial infarction, and found to predict both worsened morbidity and mortality [2,3]. TI is defined as the sum of isovolumic contraction and relaxation times divided by the ejection time, and calculated as shown in Figure 1. In systemic hypertension, there are conflicting reports on the type of association between TI, left ventricular (LV) morphology and hypertrophy (LVH) [4,5]. The present study therefore set out to describe the nature of this relationship if any, and to also assess whether a relationship exists between the geometric patterns and LV ejection fraction (LVEF) so as to establish a basis for comparison.

## Methods

The study was carried out in the echocardiography laboratory of Aminu Kano Teaching Hospital (AKTH) in Kano, North-Western Nigeria.

The Research Ethics Committee of the Hospital reviewed and approved the study protocol, which conformed to the ethical guidelines of the Declaration of Helsinki, on the principles for medical research involving human subjects [6]. The study was cross-sectional in design. Hypertensive subjects referred for echocardiography to AKTH were serially recruited from October 2008 to September 2009, after obtaining informed consent. Minimum sample size was estimated using a validated formula,[7] applying a prevalence of hypertensive heart disease (HHD) in Kano of 56.7% (among patients referred for echocardiography),[8] and a sample error of 10%.

All the recruited subjects were hypertensive in sinus rhythm and on treatment, whether or not blood pressure (BP) was controlled. Subjects with other conditions that could cause LVH or myocardial diseases were excluded, such as ischemic heart disease (IHD), valvular heart disease and cor-pulmonale. IHD was excluded if all of the following were absent: history of angina or IHD, electrocardiographic changes suggestive of myocardial infarction, and regional wall motion abnormalities on echocardiography.

Transthoracic echocardiography was performed by the author and Colleagues using Aloka Cardiac Ultrasound System (model SSD 4000 PHD), and the procedures were carried out according to standard guidelines [9]. Left ventricular ejection fraction (LVEF) was calculated using Teicholz's M-mode formula while LV mass index (LVMI) was calculated using Devereux's formula [10,11].

In adults, values of LV TI < 0.40 were considered normal, while higher values were considered abnormal, as they were found to correspond to worse pathological states, and to overall cardiac dysfunction [3].

The four patterns of LV geometry were classified and defined as: normal geometry (normal LV relative wall thickness (RWT) and LVMI); concentric remodelling (increased LV RWT with normal LVMI); concentric LVH (increased LV RWT and LVMI); and eccentric LVH (normal LV RWT with increased LVMI). RWT was calculated as: RWT = 2(LV posterior wall thickness at end-diastole)/LV end-diastolic dimension [12]. Increased LVMI was defined as values > 125 g/m^2 ^for all subjects [12].

Data were analysed with SPSS version 16.0. Means and standard deviations were computed and presented for quantitative variables. Student's *t*-test, Fisher's exact and Chi square (χ^2^) tests were used for comparison between groups as appropriate. Binary logistic regression models, and Pearson's Correlation (r) Coefficient, were used to analyse the associations between TI or LVEF and a number of variables. A p-value < 0.05 was regarded as significant.

## Results

A total of 142 subjects were recruited into the study, and the results presented in Tables 1, 2 and 3 respectively. The mean age for all patients was 56.89 ± 6.90 years, mean TI was 0.311 ± 0.219, while their mean LVEF was 53.61 ± 19.20%. The prevalence of abnormal TI (26.8%; 38 persons) in the total population of 142 persons was lower than that of reduced LV ejection fraction (< 50%) (38.0%; 54 persons), but the difference was not statistically significant (p = 0.335). LV end-diastolic dimension (LVEDD) and heart rate (HR) were the only variables found to be significantly different between subjects with normal and those with abnormal TI. In a univariate analysis, HR was the only variable that was significantly associated with TI with the following statistics: B = -0.002; standardised regression coefficient = -0.212; 95% confidence interval (CI) = -0.004 - < 0.0001; p = 0.014. When HR and LVEDD were tested in a multiple linear regression model, HR remained a significant predictor of TI with regression coefficient of -0.218, 95% CI of -0.005 - < -0.001 and p-value of 0.011. Similarly, HR was the only variable that significantly predicted abnormal TI in a binary logistic regression model with an odds ratio (OR) of 1.058 (95% CI = 1.002-1.118; p = 0.044), and also the only variable that correlated with TI significantly (correlation coefficient (r) = -0.212; p-value = 0.014). There was no relationship between TI and indices of LV diastolic dysfunction.

**Table 1:** Baseline characteristics of subjects

Variables	Normal Tei Index N = 104	Increased Tei Index N = 38	p-value
Tei Index	0.203 ± 0.102	0.605 ± 0.177	< 0.001*
Age(years)	58.04 ± 16.56	53.76 ± 17.63	0.183
M:F ratio	47:57	20:18	0.454
SBP(mmHg)	155.07 ± 29.80	155.61 ± 30.35	0.927
DBP(mmHg)	94.08 ± 20.36	96.50 ± 19.66	0.540
HR/min	94.05 ± 18.88	86.12 ± 16.50	0.031*
Diabetes Mellitus	11(10.6)	2(5.3)	0.514
NYHA	2.30 ± 1.14	2.03 ± 1.23	0.209
P/Oedema	36(34.6)	11(29.0)	0.554
LA(mm)	39.47 ± 7.78	38.76 ± 6.77	0.621
LVEDD(mm)	52.06 ± 10.20	56.39 ± 14.01	0.045*
LVEF%	54.95 ± 19.07	49.95 ± 19.34	0.170
LVEF < 50%	37(35.6)	17(44.7)	0.335
E:A ratio	1.35 ± 1.14	1.46 ± 1.39	0.707
E:e' ratio	4.08 ± 2.08	4.58 ± 2.22	0.309

Key: M:F, Male:Female; SBP and DBP, systolic and diastolic blood pressures respectively; NYHA, New York Heart Association classification of heart failure symptoms; p/oedema, peripheral oedema; LA, Left Atrium; LVEDD, LV end-diastolic dimension; LVEF, left ventricular ejection fraction; MV, mitral valve; E:A, ratio of early to late peak filling velocities; e', early diastolic mitral annular velocity; *, p-value statistically significant. All values were expressed as means ± standard deviations, or as numbers with percentages in parentheses.

**Table 2:** Association between Tei Index and LV geometric patterns.

LV geometry	Normal TI N = 104	Increased TI N = 38	OR	95% CI	p-value
Eccentric LVH	35(33.7)	19(50)	0.614	0.209-1.807	0.076
Concentric LVH	29(27.9)	9(23.7)	1.074	0.327-3.526	0.617
Concentric LV remodelling	22(21.2)	4(10.5)	1.833	0.448-7.511	0.147
Normal LV geometry	18(17.3)	6(15.8)	0.897	0.302-2.412	0.831

Key: Some of the values were expressed as numbers with percentages in parentheses.

**Table 3:** Association between LVEF < 50% and LV geometric patterns.

LV geometry	OR	95% CI	p-value
Eccentric LVH	10.46	4.781-23.870	< 0.001
Concentric LVH	0.409	0.168-0.936	0.033
Concentric LV remodelling	0.378	0.130-0.089	0.046
Normal LV geometry	0.054	0.003-0.305	< 0.001

The result also showed that there was no association between any LV geometric pattern and abnormal TI. There was also no relationship between LVH (eccentric LVH + concentric LVH) and abnormal TI (OR = 1.743; CI = 0.774-4.128; p = 0.254). This result contrasts significantly with the relationship between the geometric patterns and reduced LVEF (< 50%); tested in a binary logistic regression model and shown in Table 3. Further analysis showed that HR was higher in subjects with LVEF < 50% (97.98 ± 19.77/min) as compared with those who had LVEF≥50% (88.55 ± 16.20/min) (p = 0.004). In a univariate regression analysis, assessing the relationship between LVEF and HR, the regression coefficient was -0.253, CI -0.440 to -0.089 and p-value was significant at 0.003. LVEF also correlated negatively with HR (r = -0.253; p = 0.003) and positively with systolic blood pressure (SBP) (r = +0.240; p = 0.005), but not with diastolic BP (DBP) (r = +0.108; p = 0.218).

Subjects with normal and increased TI were on similar antihypertensive drugs, including angiotensin converting enzyme inhibitors, angiotensin II receptor blockers, beta-adrenoceptor blockers, calcium channel blockers and thiazide diuretics. The mean systolic BP for all patients was 155.22 ± 29.83 mmHg, while their mean diastolic BP was 94.74 ± 20.13 mmHg.

## Discussion

This study has found that LV geometric patterns as defined by Ganau et al [12], LVH and LVEF were not associated with TI in hypertensives, but there were strong relationships between all LV geometric patterns and LVEF. The lack of relationship between TI and LV morphology could be related to how it is obtained; it is calculated from time intervals and not dimensions as shown in Figure 1. In contrast, the Teicholz's formula is calculated from LV dimensions and assumes that the LV is ellipsoid in morphology; a possible explanation for its relationship with LV geometry [10]. The results also showed that the prevalence of abnormal TI in the present study tended to be lower than that of low LVEF, though the differences were not statistically significant. When compared with LVEF estimation using radionuclide angiography, the Teicholz's formula was found to overestimate LVEF by 10% in the presence of LVH [13]. The present study therefore lends support to the finding that estimated LVEF using Teicholz's formula could be unreliable in the presence of abnormal LV morphology [13,14]. Despite the comments above, subjects with abnormal TI correspondingly had higher prevalence of reduced LVEF and lower mean LVEF, as compared with those who had normal TI, although p-values were not statistically significant. The tendency for these indices to agree is important given that both are widely used to assess a similar outcome, which is ventricular performance or function.

We also found that TI was dependent on HR, which does not agree with some earlier reports that suggested that TI is independent of HR [1]. That TI is dependent on HR is conceivable given that numerous studies have shown that systolic and diastolic time intervals are closely linked to systolic and diastolic left ventricular performance [3]. Masugata et al similarly reported that HR was the only clinical parameter that correlated with TI in a study on hypertensive patients (r = +0.164, p < 0.05) [5]. In contrast, we observed that TI correlated negatively and significantly with HR (r = -0212; p = 0.014), which explains why HR was significantly higher in subjects with lower TI. This observation could not have been due to the effect of use of beta-blockers or digoxin, because the pattern of prescriptions was similar between the 2 compared groups. To strengthen the argument that this is an inherent characteristic and not due to haemodynamics, blood pressures and mean NYHA in the 2 groups were not significantly different. However, LVEF correlated negatively with HR (r = -0.253; p = 0.003) and positively with SBP (r = +0.240; p = 0.005), suggesting that their relationship is dependent on haemodynamics. Large prospective studies are needed to explore these relationships further.

A cross-sectional study carried out in Osogbo, south-western Nigeria, has just been published [4]. The main aim of the study was to describe the relationship between LV geometry and TI in 164 hypertensive patients. The subjects were similar to those in the present study in terms of their mean age (56.6 ± 12.5 years), male:female ratio (1.1:1) and mean blood pressures (147.9 ± 24.0/89.9 ± 11.7 mmHg). However the result for HR of the subjects was not presented, hence the relationship between TI and HR was not assessed. The results of this study contradict with ours in several respects. Firstly, the most prevalent LV geometric pattern was concentric LVH (41.4%) while eccentric LVH was the most prevalent geometric pattern (38.0%) in our study. Secondly, the authors reported that LVEF, LV shortening fraction (LVSF) and MV E:A ratio were independent predictors of TI, while LV internal dimension at end-systole, LVEF, LVSF and MV E:A ratio all correlated significantly with TI. However, they reported that there was no relationship between TI and LV geometry, in agreement with our result. In a different study, Masugata et al reported that LVEF and LVMI were not associated with TI, while Yilmaz et al reported that TI was associated with indices for LV geometry (LV mass index and RWT) [5,15].

From the foregoing therefore, it could be said that there are conflicting reports on the relationship between TI and clinical and echocardiographic variables. To sort out these conflicts, large prospective studies are needed which should also assess the impact of TI on all-cause and cardiovascular mortality.

Aside from the estimation of TI, tissue Doppler imaging has several other clinically-applicable roles. These include assessment of ventricular diastolic dysfunction even in the presence of atrial fibrillation, diagnosis of acute myocardial ischemia, ventricular long-axis systolic function, and more recently in assessing mechanical ventricular dyssynchrony [16-19].

The limitations of the present study include those inherent to the TI,[3] such as the lack of ability of TI to distinguish between grades of severity of diastolic dysfunction, its partial dependence on preload, and lack of evidence in its support in large scale epidemiologic studies. Still it is easy to obtain and is reproducible without significant inter-observer variability, and gives information on global ventricular myocardial performance. Another limitation is the use of Teicholz's formula to estimate LVEF, which also has the inherent tendency to over-estimate it in the presence of abnormal LV geometry. To minimise this inaccuracy of the Teichholz's formula, subjects with regional wall motion abnormality were excluded from the present study. The formulae that estimate LV mass using measurements obtained from 2D-guided M-mode echocardiography have several limitations, including sub-optimal accuracy in the presence of abnormal LV geometry, large inter-observer variability and poor inter-study reproducibility [20]. To minimise this, 87% of the echocardiograms were carried out by the author (KMK), while the remaining 13% were carried out by my colleagues (SM and NS) largely under my supervision. My intra-observer variability is about 4.3%, but we are in the process of estimating the inter-observer variability for our echocardiography laboratory.

## Conclusion

This study has found that LV geometric patterns, LVH and LVEF were not associated with TI in hypertensives, but there were strong relationships between all LV geometric patterns and LVEF. The lack or existence of a relationship between TI or LVEF respectively, and LV morphology, could possibly be related to whether or not they depend on LV dimensions in their determination. In addition, the study found that TI was dependent on HR, in agreement with some earlier reports. The results of the present study have further clarified the relationship between a measure of ventricular performance (TI) and LV geometric patterns, as well as their relationships with LVEF.

## Competing interests

The author declares that they have no competing interests.

## Authors' contributions

The author (KMK) conceived and carried out the study, analysed data, and wrote and approved the manuscript.
